# Peripapillary Intrachoroidal Cavitation in Myopia Evaluated with Multimodal Imaging Comprising “En-Face” Technique

**DOI:** 10.1155/2015/890876

**Published:** 2015-10-12

**Authors:** Georges Azar, Romain Leze, Aude Affortit-Demoge, Céline Faure

**Affiliations:** ^1^Eye & Ear University Hospital, Faculté de Médecine, Université Saint-Esprit de Kaslik, P.O. Box 70-933, Beyrouth, Lebanon; ^2^Fondation Ophtalmologique Adolphe de Rothschild, 25 rue Manin, 75019 Paris, France; ^3^Hôpital Privé Saint Martin, 18 rue des Roquemonts, 14050 Caen Cedex, France

## Abstract

*Objectives*. To demonstrate the usefulness of “en-face” Spectral Domain Optical Coherence Tomography (SD-OCT) combined with Fluorescein Angiography (FA) in the investigation of peripapillary intrachoroidal cavitation. *Materials and Methods*. A 72-year-old man followed for primary open-angle glaucoma (POAG) for 4 years was referred for an asymptomatic “peripapillary lesion.” A full ophthalmological examination and conventional imaging of the retina were done. FA, Indocyanine Green Angiography (ICG-A), and SD-OCT using the “en-face” technique were also performed. *Results*. Best-corrected visual acuity (BCVA) was 20/25 both eyes. Slit-lamp examination revealed no abnormalities of anterior segment. Intraocular pressure (IOP) was normal. Fundus examination showed a triangular yellow-orange thickening at the inferior border of both optic nerves. FA showed early hypofluorescence of the lesion and progressive staining without any dye pooling. SD-OCT with “en-face” technique showed an intrachoroidal hyporeflective space resembling a cavitation below the retinal pigment epithelium (RPE). *Conclusions*. “En-face” SD-OCT and FA are valuable techniques for the diagnosis of peripapillary intrachoroidal cavitation associated with myopia. Pathophysiological insights regarding SD-OCT findings and angiography behavior are offered.

## 1. Introduction

The peripapillary detachment of the retinal pigment epithelium (RPE) and retina is one of the disorders that may be observed in myopic eyes. It was first described by Freund et al. [1] on Optical Coherence Tomography (OCT) as a localized RPE detachment around the optic disc. They also called it peripapillary detachment in pathologic myopia (PDPM). Toranzo et al. have renamed this abnormality “peripapillary intrachoroidal cavitation,” finding that this lesion was located inside the choroid and that the underlying RPE and retina were normal [2].

“En-face” OCT imaging is a novel technology that enhances sensitivity in the detection and follow-up of many disorders of the posterior pole. It allows earlier detection and offers deep insight into the understanding of mechanisms underlying retinal lesions [3–5]. We hereby present a case of peripapillary intrachoroidal cavitation and describe the features found on Spectral Domain Optical Coherence Tomography (SD-OCT) using the “en-face” technique. We also present a pathophysiological explanation for the underlying features found on Fluorescein Angiography (FA) and Indocyanine Green Angiography (ICG-A). To the best of our knowledge, no explanation has been offered in the literature regarding the angiographic behavior.

## 2. Case Report

**Figure 1:**
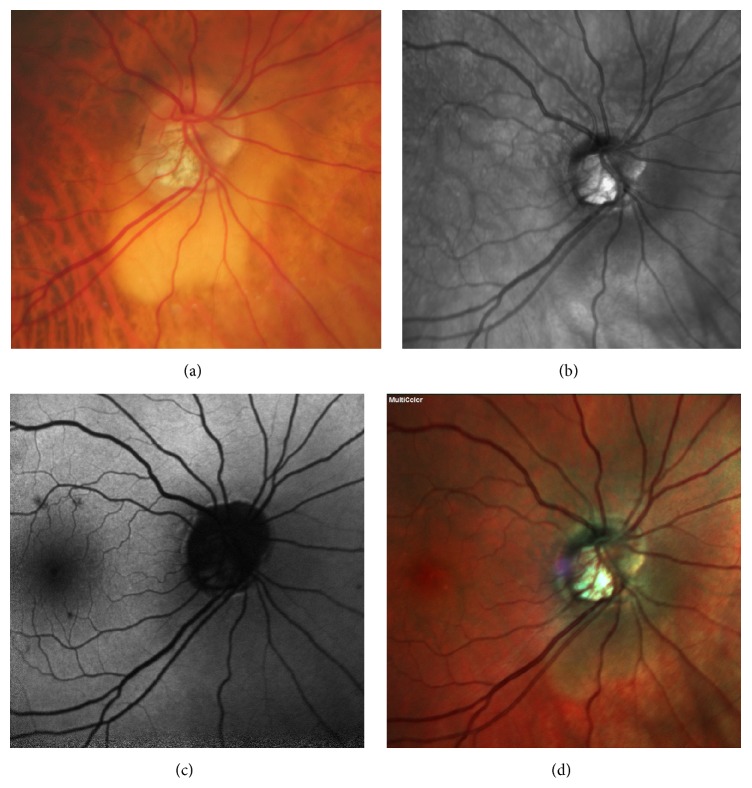
Peripapillary intrachoroidal thickness and cavitation. Color fundus photograph (a), infrared imaging (b), autofluorescence imaging (c), and multicolor fundus picture (d) showing a well-circumscribed enlargement of the yellow-orange peripapillary area, at the inferior border of the myopic conus.

A 72-year-old man was referred for a peripapillary “lesion” evaluation. He has been followed for a primary open-angle glaucoma (POAG) for the past 4 years and treated with latanoprost 1 drop daily. His medical history was unremarkable. At presentation, his best-corrected visual acuity (BCVA) was 20/25 in both eyes with the following refraction: −2.00 (−1,00 × 90°) in the right eye (OD) and −2.50 (−1,00 × 100°) in the left eye (OS). Slit-lamp examination revealed normal anterior segment in both eyes, and the intraocular pressure (IOP) taken with Goldmann applanation was 11 mmHg OD and 12 mmHg OS. Fundus examination revealed a well-circumscribed triangular yellow-orange thickening at the inferior border of both optic nerves (Figure 1).

**Figure 2:**
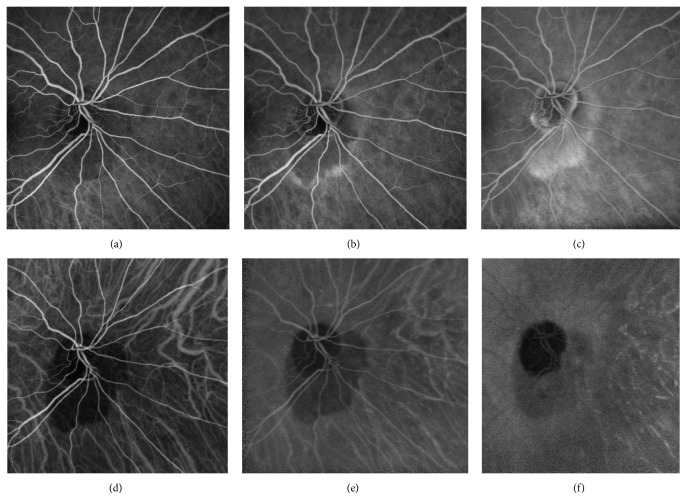
Fundus Fluorescein Angiography ((a) early stage at 1 minute, (b) intermediate stage at 3 minutes, and (c) late stage at 8 minutes) showing early hypofluorescence of the lesion with progressive* late* staining without any dye pooling. Indocyanine Green Angiography ((d) early stage at 2 minutes, (e) intermediate stage at 10 minutes, and (f) late stage at 20 minutes) showing the peripapillary cavitation to be hypofluorescent throughout the entire sequence.

**Figure 3:**
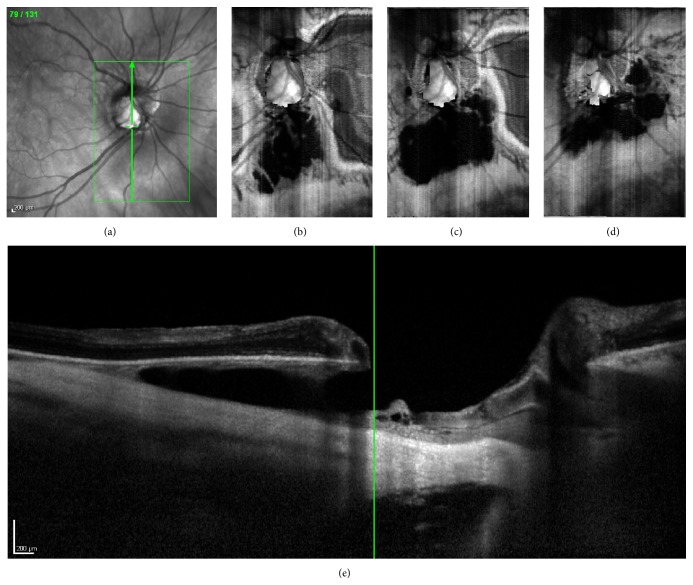
Infrared imaging (a) with localization of B-scan acquisition within the peripapillary cavitation (green arrow). “En-face” Spectral Domain Optical Coherence Tomography (SD-OCT) (b, c, and d) and vertical B-scan through the peripapillary cavitation (e), showing a large intrachoroidal cavitation space separating the retinal pigment epithelium (RPE) from the sclera. Note the lateral “V-shaped” extension of the cavitation inside the choroid on (e).

Visual field (VF) examination performed with the Humphrey Field Analyzer (Carl Zeiss Meditec, Dublin, CA) showed an enlargement of the blind spot in both eyes, a superior arcuate scotoma in the right eye, and a nasal bridge in the left eye. Both blind spot areas seemed to have grown larger than those shown in the previous VF exams, which were performed yearly for 4 years. FA (Heidelberg Retina Angiograph Heidelberg Engineering, Heidelberg, Germany) showed early hypofluorescence with progressive staining of the peripapillary lesion. This staining remarkably increased during the late sequences. No dye pooling was noted during all FA sequences (Figures 2(a)–2(c)). On the ICG-A (Heidelberg Retina Angiograph Heidelberg Engineering, Heidelberg, Germany), the peripapillary cavitation was hypofluorescent throughout the entire sequence (Figures 2(d)–2(f)). SD-OCT (Heidelberg Engineering, Heidelberg, Germany) with “en-face” technique, combined with vertical scans passing through the peripapillary lesion area, showed a large intrachoroidal hyporeflective space, resembling a cavitation separating the RPE from the sclera adjacent to the optic nerve head. A lateral “V-shaped” extension of the cavitation inside the choroid, which seems to be extremely thin around the excavation, is shown as well (Figure 3).

## 3. Conclusion

New current SD-OCT modalities have led to a better understanding of many retinal diseases [6]. Peripapillary intrachoroidal cavitation is a yellow-orange peripapillary abnormality that may be found in high myopia [1, 2]. Contrary to the common belief, this lesion seems to be common in myopic eyes and has to be differentiated from glaucoma [7, 8], due to the possible presence of multiple glaucoma like visual field defects, some optic disk rotational changes around both the vertical and sagittal axis [9], and finally the possible repercussions on retinal nerve fiber layer measurements [10].

To better illustrate the pathophysiology underlying this peripapillary lesion, the different phases during FA were interpreted in conjunction with “en-face” technique SD-OCT. Early hypofluorescence may be explained by the extremely thin choroid regarding the lesion, as measured with SD-OCT with “en-face” technique, compared to adjacent normal choroid, which makes it less stained by fluorescein during early phases. Progressively, especially during late phases, the lesion seems to have the same impregnation with fluorescein as the adjacent choroid, due to the late scleral impregnation through the thin choroid.

In conclusion, “en-face” SD-OCT combined with FA is valuable technique for the diagnosis of peripapillary intrachoroidal cavitation associated with myopia. Both combined, they allow a thorough pathophysiological explanation and deep insights regarding SD-OCT findings and angiography behavior during the different sequences.
